# Engulfment of Activated Apoptotic Cells Abolishes TGF-β–Mediated Immunoregulation via the Induction of IL-6

**DOI:** 10.4049/jimmunol.1401256

**Published:** 2015-01-19

**Authors:** Clare A. Notley, Mark A. Brown, Jenny L. McGovern, Christine K. Jordan, Michael R. Ehrenstein

**Affiliations:** Division of Medicine, Centre for Rheumatology, University College London, London WC1E 6JF, United Kingdom

## Abstract

Phagocytosis of apoptotic cells (ACs) is usually a potent immunoregulatory signal but can also promote inflammation. In this article, we show that administration of apoptotic dendritic cells (DCs) inhibited inflammation in vivo through increasing production of TGF-β from intrinsic DCs and B cells. However, ACs derived from LPS-activated DCs failed to restrain inflammation because of a short-lived but marked IL-6 response, which abolished the increase in TGF-β. Inhibition of IL-6 restored the protective anti-inflammatory properties of aACs and the TGF-β response. DCs isolated from mice that had received resting but not activated ACs could transfer the suppression of inflammation to recipient mice. These transferred DCs stimulated B cell TGF-β production and relied on an intact B cell compartment to limit inflammation. These results highlight how the activation state of AC governs their ability to control inflammation through reciprocal regulation of IL-6 and TGF-β.

## Introduction

The rapid clearance of apoptotic cells (ACs) by phagocytes plays a central role in maintaining tissue homeostasis and immune tolerance to self-Ags (1–3). Defects or disruption to AC clearance can lead to the chronic accumulation of apoptotic material, the initiation of inflammation, and ultimately the development of autoimmunity or persistent inflammatory disease (4, 5). Macrophages, dendritic cells (DCs), and B cells all contribute to maintaining an immunosuppressive environment during the engulfment of ACs via their production of IL-10 and TGF-β (6–10).

However, ACs are not always tolerogenic and can instead promote further inflammation depending on their past experiences (4). For instance, ACs generated during infection can alter their normally regulatory effects through generation of inflammatory cytokines and the induction of Th17 cells (11). Apoptotic cancer cells are even more immunogenic than necrotic cells (12), and when cross-presented by DCs, they induce strong CTL responses (13, 14). Indeed, apoptotic cancer cells have been trialed for use in cancer cell vaccines aimed at boosting immunity to cancer Ags (15, 16). Expression of CD40L by apoptotic T cells has been shown to induce DC maturation and cross priming of CTL responses (17). The source of the AC and the phagocytic cell involved can also influence the immune response. Thus, apoptotic thymocytes and splenocytes induce an IL-10–rich, suppressive environment when engulfed by splenic cells (10, 18), whereas apoptotic DCs phagocytosed by resident DCs support tolerance via the production of TGF-β (19, 20).

In this report, we provide evidence that ACs induced from previously activated DCs preferentially increase IL-6 levels, whereas TGF-β production by DCs and B cells is promoted by resting ACs, thereby limiting inflammation. Reciprocal regulation of TGF-β and IL-6 by AC appears to determine the balance between inflammation and tolerance.

## Materials and Methods

### Mice

C57BL/6J mice and μMT mice (Jackson Laboratories, Sacramento, CA) were bred and maintained in specific pathogen-free facilities under home office guidelines. Mice were used between 8 and 14 wk of age. All experiments were approved by the Ethical Review Committee.

### Preparation of ACs

DCs were differentiated from the bone marrow of mice by culture for 5 d with GM-CSF (Peprotech, Rocky Hill, NJ). On day 5, DCs were either left untreated or stimulated overnight with 1 μg/ml LPS (Sigma-Aldrich, St. Louis, MO). DCs were then cultured with 10 μM etoposide (Sigma-Aldrich) for 5 h, and the level of apoptosis was determined by Annexin V and propidium iodide (PI) staining. Typically, both unstimulated ACs and activated ACs (aACs) were between 60 and 75% Annexin V^+^ and 8 and 11% PI^+^. In some experiments, DCs were stained with 1 μM PKH-26 (Sigma-Aldrich) before activation and induction of apoptosis.

### Induction of arthritis

Mice were immunized with methylated BSA (mBSA; Sigma-Aldrich) as described previously (18, 21). Some mice received 20 × 10^6^ ACs or aACs i.v. for 3 consecutive days after immunization. Other groups of mice received 1.5 × 10^6^ DCs at the time of immunization. In some experiments, neutralization of IL-6 occurred at the time of AC transfer with i.p. injection of 100 μg LEAF purified anti-mouse IL-6 Ab (BioLegend), or control mice were treated with rat IgG1 isotype control (BioLegend). Clinical score was determined by the degree of limping, where 0 = normal walking, 1 = mild limping, 2 = severe limping, and 3 = unable to put weight on leg (18). Day 3, 6, or 7 post intra-articular injection, inguinal draining lymph nodes and spleens were removed and stimulated with anti-CD3 mAb (0.1 ng/ml) for 72 h. Supernatants from cultures were tested for IL-17A and IL-10 by ELISA (R&D Systems, Minneapolis, MN). In some experiments, ACs or aACs were transferred on the day of immunization only, and spleens were harvested and analyzed 48 h later.

### Confocal microscopy

A total of 500,000 thioglycollate-elicited peritoneal macrophages were cultured with 2.5 × 10^6^ ACs or aACs for 5 h on glass coverslips in the presence of GolgiStop. Cells were washed with PBS and cell dissociation buffer, and fixed for 10 min with methanol. Cells were stained with anti–IL-10 PE, anti-TNF PE, or rat IgG PE isotype control (1:25 dilution in PBS containing 2% BSA) at 4°C overnight. Cells were washed with PBS-Tween, and mounted and analyzed using a Leica TSC SPE confocal microscope (Leica, Buffalo Grove, IL).

### In vivo engulfment

Unstimulated or LPS-stimulated, PKH-26–labeled DCs were induced to undergo apoptosis, washed, and i.v. injected into wild type mice. One, 2, or 4 h after injection, spleens were harvested and half frozen for RNA isolation and half used for flow cytometry staining. Splenocytes were stained for CD19, CD11c, and F4/80, and the cells responsible for AC or aAC engulfment were determined. In some experiments, spleens taken at 2 h after injection were stained and analyzed by ImageStream. PKH-26 cells costaining with CD11c, CD19, or F4/80 were analyzed to determine whether they were engulfed ACs or attached to the surface of splenic cells.

### In vitro engulfment

To test whether IL-6 production from phagocytes engulfing aACs was due to carryover LPS, using 40 ng/ml M-CSF (Peprotech), we left macrophages derived from bone marrow untreated or pretreated for 5 h with 3 μM TLR4 inhibitor CLI-095 (Invivogen, San Diego, CA). Macrophages were then cultured for 72 h with ACs or aACs, and supernatants were taken to determine IL-6 concentration by ELISA (eBioscience).

### TaqMan RT-PCR

RNA was isolated from spleens using TRIzol reagent (Invitrogen, Grand Island, NY) and Precellys homogenization tubes (Peqlab, Sarisbury Green, U.K.). cDNA was transcribed from 1 μg RNA using a reverse transcription kit (Qiagen, Valencia, CA), and TaqMan RT-PCR was performed in 96 wells using an ABI PRISM detection system. TaqMan primers and probes (Applied Biosystems, Foster City, CA) were used to detect the expression of IL-6, IL-10, TNF-α, TGF-β, and HPRT (endogenous control). Data were analyzed using the comparative threshold cycle (CT) method, normalizing data to HPRT and shown as fold change relative to the no AC (untreated) mice.

### Flow cytometry

Cells required for intracellular cytokine staining were cultured for 6 h in RPMI 1640 containing PMA (Sigma-Aldrich) and ionomycin (Sigma-Aldrich). GolgiStop (BD Biosciences) was added to the culture for the last 4 h. Cells were surface stained with anti–CD19-PeCy7 or Pacific Blue (BioLegend), anti–TGF-β–PE (R&D Systems), anti-CD11c FITC or allophycocyanin (eBioscience), or anti-F4/80 allophycocyanin (eBioscience). Cells were then washed and stained with anti-mouse IFN-γ PE, anti-mouse IL-17 Alexa Fluor 647 (eBioscience), or anti-mouse IL-10 PE and analyzed by flow cytometry. Where possible, all gates were set using isotype control Abs. All samples were run on the LSRII or LSR Fortessa and analyzed using FlowJo software (Tree Star, Ashland, OR). All Abs were purchased from BD Biosciences unless otherwise stated.

### Cytokine bead array

DCs, macrophages, and B cells were sorted from the spleens of wild type mice using the FACSAria (BD Biosciences) based on expression of CD11c, F4/80, and CD19, respectively. A total of 200,000 cells were cultured for 24 h with 400,000 ACs or aACs at 37°C and 5% CO_2_. Supernatants were collected and 50 μl was analyzed for IL-6, TNF-α, and IL-10 using cytokine bead array (BD Biosciences).

### DC transfer

Forty-eight hours after AC injection, spleens were harvested and DCs isolated using CD11c microbeads, following the manufacturer’s instructions (Miltenyi Biotech, Auburn, CA). Purities of CD11c^+^ cells ranged from 83 to 95%. DCs were adoptively transferred i.v. into wild type or μMT mice at the time of immunization with mBSA/CFA. In some experiments, mice were treated i.p. with 400 μg TGF-β R1 kinase inhibitor VI (Millipore, MA) every 3 d from the day of DC transfer.

### Statistical analysis

All data were analyzed using GraphPad Prism software (San Diego, CA). The statistical analyses performed are noted with the corresponding data. In general, data were analyzed by one-way ANOVA followed by Bonferroni post hoc testing for multiple comparisons or paired or unpaired *t* test.

## Results

### Resting and aACs differentially regulate the production of anti-inflammatory and proinflammatory cytokines

We first sought to identify a system in which we could test and compare the ability of resting and activated cells that have undergone apoptosis to induce a differential cytokine response by phagocytes during clearance. Resting (ACs) and LPS aACs were therefore generated from bone marrow–derived DCs. DC activation was associated with an upregulation of CD80, CD86, and MHCII expression compared with resting DCs. LPS activation of DCs did not upregulate CD40L, nor did it affect the apoptotic phenotype (determined by Annexin V and PI staining; data not shown) of the aACs compared with the ACs after culture with etoposide to induce apoptosis.

**FIGURE 1.**
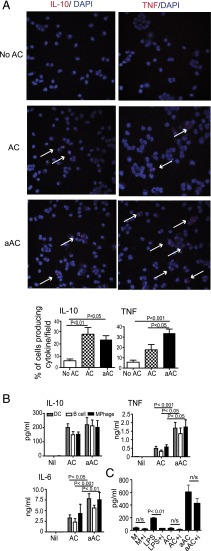
aACs induce production of the proinflammatory cytokines IL-6 and TNF-α in vitro. (**A**) Peritoneal macrophages were cultured for 6 h alone (No AC) or with apoptotic DCs (AC) or LPS-activated apoptotic DCs (aAC) and then stained for IL-10, TNF-α (red), and DAPI (blue) and imaged using a Leica TSC SPE confocal microscope (original magnification ×40). Arrows show cytokine production. Bar charts show combined data of the mean ± SEM percentage of cytokine-producing cells per field from six independent experiments. (**B**) Splenic DCs, B cells, and macrophages were cultured for 24 h alone (Nil) or with ACs or aACs. Supernatants were collected, and IL-10, TNF-α, and IL-6 levels were determined. Graphs show mean ± SEM of pooled data from three independent experiments. (**C**) Bone marrow–derived macrophages were left untreated (M) or pretreated with a TLR4 inhibitor (i). Macrophages were subsequently cultured with 0.01 μg/ml LPS, ACs, or aACs for 72 h, and IL-6 concentration was determined by ELISA. Graph shows mean ± SEM from six independent experiments.

**FIGURE 2.**
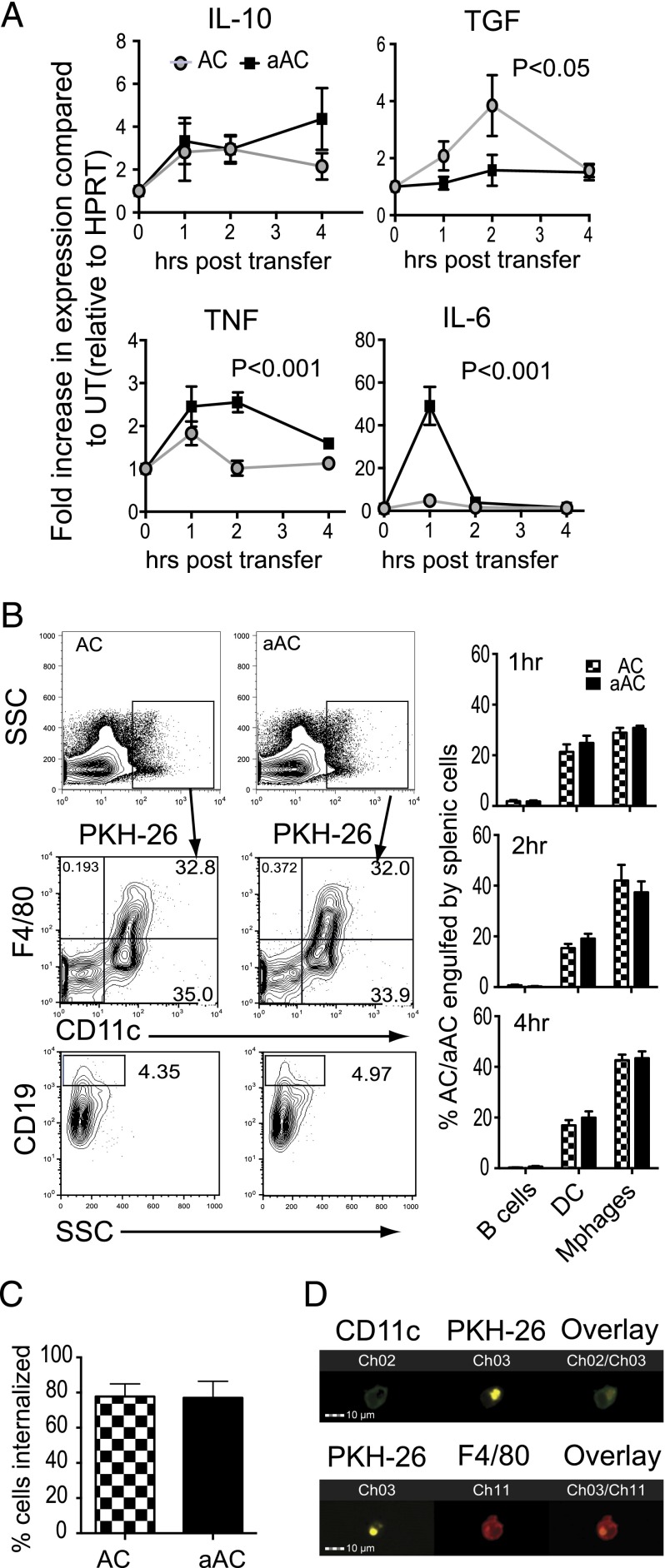
aACs induce production of the proinflammatory cytokines IL-6 and TNF-α in vivo. (**A**) C57BL/6 mice were left untreated or injected with ACs or aACs and spleens snap-frozen 1, 2, or 4 h later, and the fold increase in IL-10, TGF-β, TNF-α, and IL-6 mRNA production was determined in mice treated with ACs or aACs compared with untreated mice. All data were normalized to HPRT. Graphs show mean ± SEM of pooled data from three independent experiments. (**B**) PKH-26^+^ ACs or aACs were injected into mice, and splenic cells responsible for the engulfment of ACs and aACs were determined by gating on the PKH-26^+^ cells and analyzed for coexpression of F4/80 to identify macrophages, CD11c to identify DCs, and CD19 to identify B cells. FACS plots show representative data, and graphs show the mean ± SEM percentage of ACs and aACs engulfed by B cells, DCs, or macrophages. Data are pooled from four independent experiments. (**C**) Cells were further analyzed using ImageStream to determine the percentage of PKH-26^+^ cells engulfed in the spleen. Graph shows data from two independent experiments from six individual mice. (**D**) Images showing engulfment by DCs (*top panel*) and macrophages (*bottom panel*) are representative of the data (original magnification ×40). The markers/stains used in (D) are indicated above the lanes in the figure. Details of the fluorescent labels attached to CD11c and F4/80 are given in the *Materials and Methods* (CD11c-FITC and F4/80-APC).

To determine the cytokines induced in response to ACs or aACs, we cultured thioglycollate-elicited peritoneal macrophages (Fig. 1A) or splenic DCs, B cells, or macrophages (Fig. 1B) in vitro alone or with ACs or aACs. Whereas ACs and aACs induced equal amounts of IL-10 (Fig. 1A, 1B), aACs preferentially enhanced the production of the inflammatory cytokines TNF-α and IL-6 (Fig. 1A, 1B). To confirm that the inflammatory cytokine production was not due to LPS carryover from the cultures containing LPS, we cultured bone marrow–derived macrophages with ACs and aACs in the presence of a TLR4 inhibitor. Inhibition of TLR4 had no significant effect on the production of IL-6 by aACs but could abolish the production of IL-6 induced by stimulation with LPS (Fig. 1C). i.v. injection of 20 × 10^6^ ACs or aACs into naive mice confirmed that both aACs and ACs induced the production of IL-10 from splenocytes; however, aACs also increased the production of TNF-α and IL-6 (Fig. 2A). Notably, there was an early short burst of IL-6 production, which increased 50-fold immediately after transfer of aACs. In addition, we observed that ACs, but not aACs, induced TGF-β production during a 2-h period after transfer.

To determine whether the responses observed in vivo were due to the differential engulfment by phagocytic cells in the spleen, we transferred PKH-26–labeled ACs and aACs i.v., and the migration and engulfment of cells within the spleen were detected by flow cytometry. Engulfment of ACs and aACs, primarily by macrophages and DCs, was evident at 1, 2, and 4 h after transfer (Fig. 2B). The contribution of the various phagocytic cells within the spleen in the removal of ACs and aACs was similar, suggesting that the differences observed in cytokine production were not due to engulfment by specific cell types. Cells were also analyzed by ImageStream, confirming that ∼80% of the ACs costaining with DC or macrophage markers were internalized rather than attached to the engulfing cell (Fig. 2C, 2D).

### aACs are unable to suppress the development of inflammatory arthritis

**FIGURE 3.**
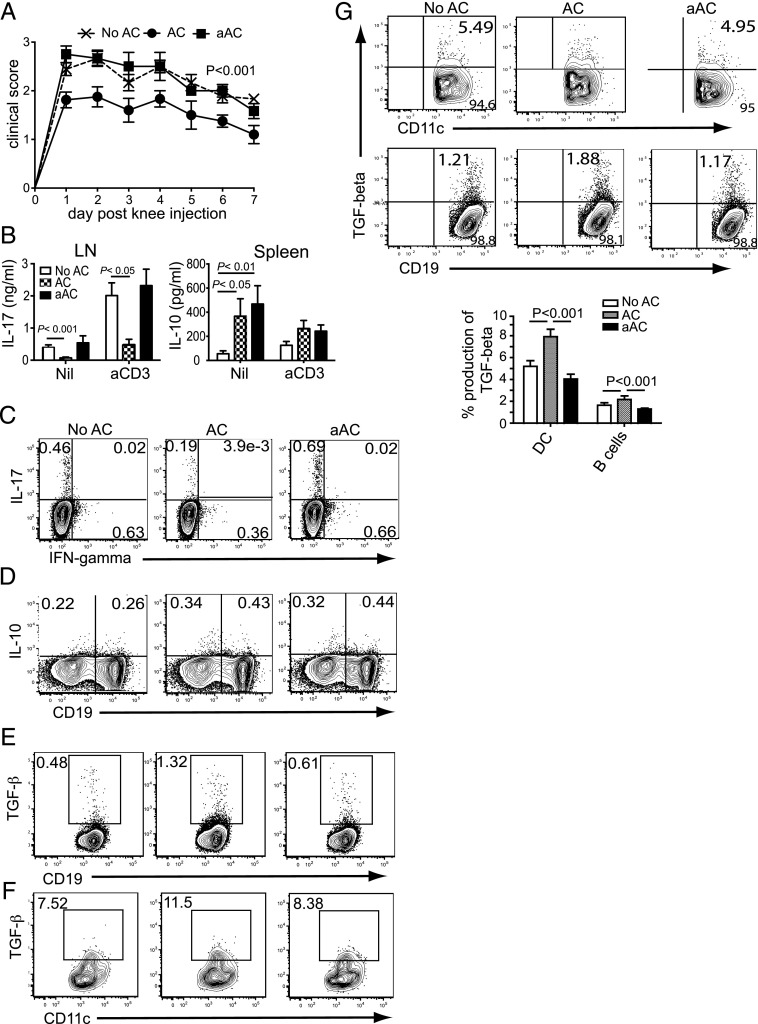
Activation of ACs abolishes the ability of ACs to induce TGF-β. (**A**) Arthritis was monitored in mice left untreated (No AC) or injected with AC or aAC on the day of immunization and for a further 2 consecutive days before intra-articular injection of mBSA to induce inflammation in the knee. Lymph nodes and spleens were harvested on day 7 after knee injection. LN cells were analyzed for IL-17 production by (**B**) ELISA after no stimulation (Nil) or stimulation with anti-CD3 mAb (aCD3) and (**C**) flow cytometry. IFN-γ production in the LNs was also determined by flow cytometry after gating on lymphocytes (C). IL-10 production by the spleen was assessed by (B) ELISA and (**D**) flow cytometry, after gating on lymphocytes. TGF-β production by splenic B cells (**E**) and DCs (**F**) was analyzed by flow cytometry. Live lymphocytes were gated; then CD11c^+^ or CD19^+^ populations were gated and assessed for TGF-β production. *n* = 12 pooled from 4 independent experiments. (**G**) Mice were left untreated (No AC) or injected with AC or aAC, then immunized and spleens harvested 48 h later. The production of TGF-β by DCs and B cells was determined by flow cytometry, gated as mentioned earlier. Histograms show pooled data from 4 independent experiments; *n* = 12. FACS plots are representative data.

It has previously been shown that transfer of thymically derived ACs at the time of immunization can suppress the development and severity of inflammatory arthritis through the production of IL-10 (10, 18). Given the differences we found with respect to cytokine production between ACs and aACs, we next sought to investigate whether both are equally potent at suppressing inflammation in vivo. We used the Ag-induced arthritis (AIA) model, which is IL-17 dependent (22, 23). Although ACs derived from resting DCs suppressed the development of AIA, activation of the DCs with LPS before apoptosis induction resulted in an inability to modulate arthritis development (Fig. 3A). ELISA (Fig. 3B) and flow cytometry (Fig. 3C–F) data show that suppression of arthritis was determined by the balance of IL-17 and TGF-β production, where decreased production of IL-17 by draining lymph node cells and an increase in TGF-β production by the splenocytes were protective. aAC transfer was neither able to suppress IL-17 responses in the lymph node nor boost TGF-β production by splenic B cells and DCs. IL-10 production by splenocytes was upregulated by aACs to a similar level observed by resting ACs (Fig. 3B, 3D).

Suppression of IFN-γ was variable between studies, consistent with a previous publication that ACs do not significantly inhibit Th1 cells during AIA (18) (Fig. 3C). Upregulation of TGF-β mRNA was observed within 4 h of AC transfer (Fig. 2A). We therefore transferred mice with 20 × 10^6^ ACs or aACs, immunized with mBSA/CFA, and analyzed the spleens 48 h later to further dissect the ability of ACs and aACs to modulate TGF-β production during the initiation of inflammation. At 48 h after transfer, a significant increase in the percentage of TGF-β–producing DCs and, to a lesser extent, B cells was observed in the spleens of AC-treated mice compared with aAC-treated mice (Fig. 3G).

### aACs are unable to suppress the development of inflammatory arthritis due to the induction of IL-6 and inhibition of TGF-β production

**FIGURE 4.**
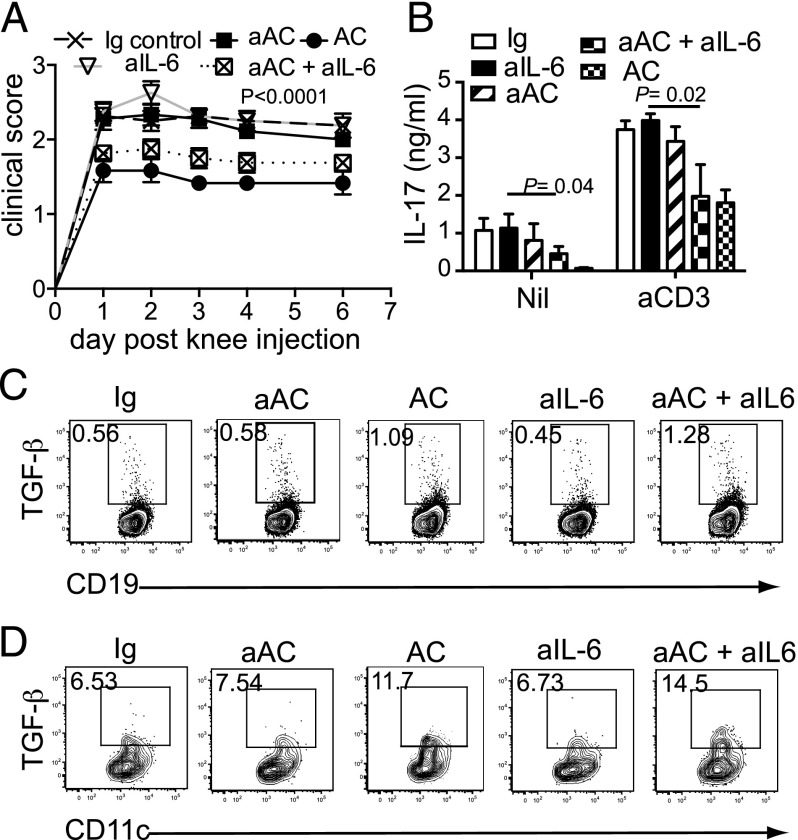
Activation of ACs abolishes their tolerogenic properties through an IL-6–dependent inhibition of TGF-β. A total of 20 × 10^6^ ACs or aACs were given i.v. to C57BL/6 mice; in some groups, aACs were given in combination with 100 μg anti–IL-6 Ab (aAC + aIL-6). Control groups were i.p. injected with 100 μg anti–IL-6 (aIL-6) or isotype control (Ig control) Abs. All groups were immunized with mBSA and CFA, and aAC, aAC + aIL-6, and AC groups received 20 × 10^6^ cells i.v. for 2 further consecutive days (**A**). Six days after knee injection, LNs were analyzed for production of IL-17 by ELISA (**B**), and splenic B cells (**C**) and DCs (**D**) were analyzed for production of TGF-β by flow cytometry. Live lymphocytes were gated; then CD11c^+^ or CD19^+^ populations were gated and assessed for TGF-β production. Data are pooled from two independent experiments; *n* = 6–8. Values in plots show the percentage of cells making the cytokine.

We next sought to determine whether shifting the balance of proinflammatory and anti-inflammatory cytokines after aAC transfer could restore the protective immune response that was observed with ACs. Because IL-6 increased greatly in the spleens of mice injected with aACs compared with ACs (Fig. 2A), we transferred aACs alone or in combination with IL-6 blockade at the time of immunization to determine the role of IL-6 in modulating immune suppression. Inhibition of IL-6 production at the time of aAC transfer resulted in suppression of AIA (Fig. 4A) and IL-17 responses (Fig. 4B), as observed after AC transfer. Furthermore, IL-6 blockade permitted the induction of TGF-β production by splenic B cells (Fig. 4C) and DCs (Fig. 4D), demonstrating that TGF-β production is inhibited by IL-6. Taken together, these results suggest that resting ACs suppress inflammation via TGF-β, whereas aACs promote IL-6 production, which blocks TGF-β production and its anti-inflammatory effects.

### TGF-β–producing DCs induced in response to resting ACs can transfer their protective effects

**FIGURE 5.**
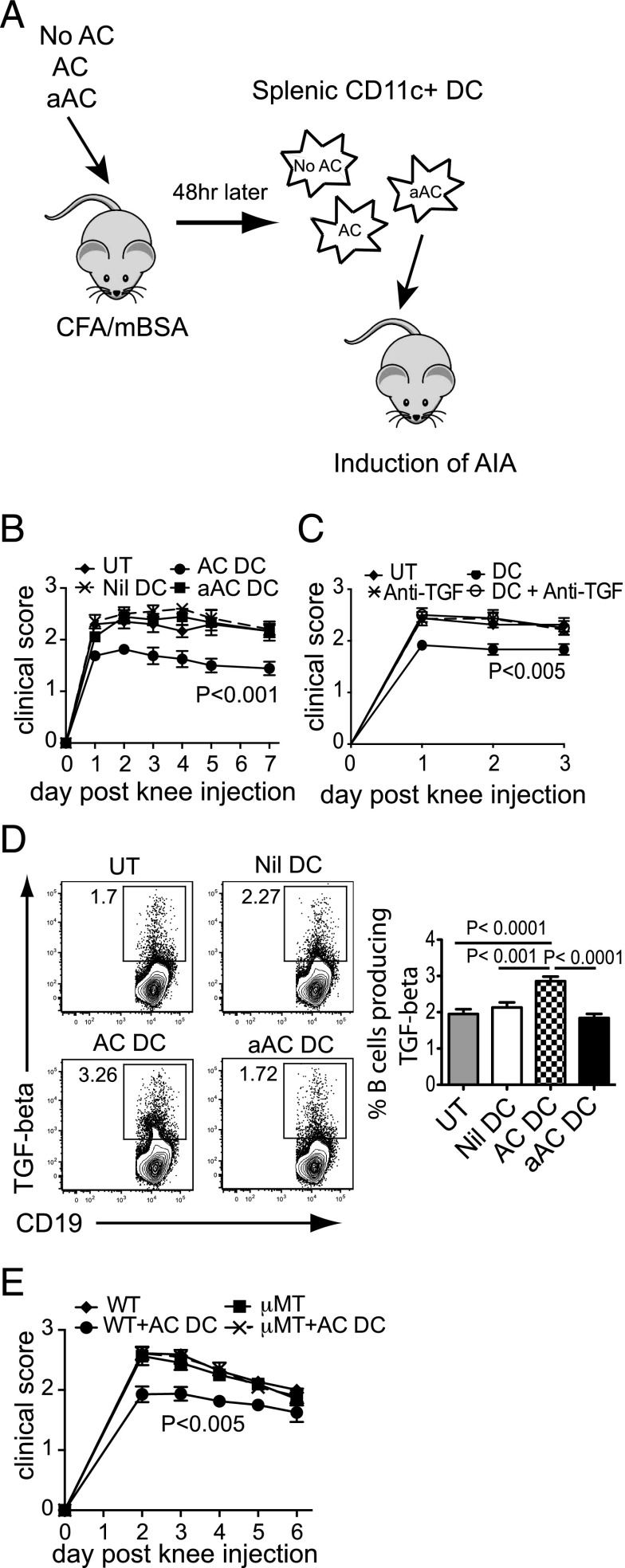
TGF-β–producing splenic DCs mediate the protective effects of ACs. CD11c^+^ DCs were isolated from the spleens of untreated (No AC) or AC- (AC DC) or aAC (aAC DC)-treated mice after 48 h; 1.5 × 10^6^ cells were transferred i.v. into naive WT mice and AIA induced as illustrated schematically in (**A**). (**B**) Clinical scores of untreated mice (UT) or mice adoptively transferred with Nil DCs, AC DCs, or aAC DCs over 7 d after arthritis induction. Graph shows pooled data from four independent experiments; *n* = 12. (**C**) AC DCs were transferred on the day of immunization in combination with TGF- β blockade using 400 μg TGF-βRI kinase inhibitor VI (DC + anti-TGF), or control mice received TGF-β blockade alone (anti-TGF). Arthritis was induced and clinical scores were determined for 3 d. Data are pooled from two independent experiments; *n* = 8. (**D**) The percentages of B cells making TGF-β from untreated mice (UT) or mice adoptively transferred with Nil DCs, AC DCs, or aAC DCs were determined 7 d after arthritis induction. FACS plots show representative data; histograms show pooled data from three independent experiments. Cells were gated for live lymphocytes, then for CD19^+^ cells. CD19^+^ cells were assessed for TGF-β production. (**E**) AC DCs were transferred into wild type (WT) or B cell–deficient mice (μMT) at the time of immunization, and clinical score was monitored for 6 d after knee injection. Data are pooled from two independent experiments; *n* = 10.

To confirm whether the ability of ACs to suppress AIA was due to the induction of TGF-β–producing splenic DCs, we isolated CD11c^+^ DCs from untreated mice and mice treated for 48 h with ACs or aACs and adoptively transferred them into C57BL/6 mice at the time of immunization with mBSA (Fig. 5A). Transfer of 1.5 × 10^6^ DCs from AC-treated mice (AC DCs) on the day of immunization was sufficient to suppress the severity of AIA. In contrast, DCs from untreated (Nil DC) or aAC-treated mice (aAC DC) were unable to modulate the disease severity (Fig. 5B). Blockade of TGF-β from the day of AC DC transfer resulted in the abrogation of the protective effects of AC DCs (Fig. 5C), demonstrating that the suppressive properties of AC DCs are mediated by their ability to produce TGF-β. Interestingly, we observed that the transfer of AC DC into wild type mice increased the production of TGF-β from B cells (Fig. 5D), as was observed after direct transfer of ACs, although the DCs were more potent inducers of B cell TGF-β than ACs (Fig. 3C). To identify a role for TGF-β–producing B cells in the suppression of arthritis, we adoptively transferred AC DCs into WT or B cell–deficient μMT mice. Suppression of disease by AC DCs was abrogated in μMT mice (Fig. 5E), suggesting that after AC transfer, TGF-β–producing DCs can, in turn, induce B cell TGF-β production and together contribute to the suppression of inflammation.

## Discussion

Maintenance of immune homeostasis and tolerance to self-Ags is dependent on the efficient disposal of ACs and the induction of an anti-inflammatory environment. Macrophages, DCs, and B cells are important in the removal of AC and the induction of immune tolerance. Although macrophages have a critical role in the engulfment and clearance of ACs (6, 7), DCs and B cells maintain tolerance, even during the onset of inflammation, via the production of TGF-β and IL-10 (18, 24, 25). Indeed, a recent study has shown that splenic metallophilic macrophages recruit regulatory T cells and DCs in response to ACs, maintaining immune tolerance to self-Ags (26). Our data extend these findings and demonstrate that resting ACs induce TGF-β production by DCs, which, in turn, drives the production of TGF-β by B cells. The consequences of ACs activated with a microbial stimulant LPS are very different, with a failure to suppress inflammation due to an early burst of IL-6 production that inhibits DC and B cell TGF-β.

A variety of receptors and associated pathways recognize and respond to cell death. The stage of cell death can influence whether a cell induces tolerance or inflammation (27). For example, the release of HMGB1 and the level of its immunological activity can be influenced by whether the cell has undergone apoptosis or necrosis. Activated macrophages can release HMGB1 in response to TLR4 activation, thereby inducing cytokine production (28). It is possible that apoptotic DCs previously activated with LPS are able to release or trigger the secretion of active HMGB1. Whether this is responsible for the IL-6 production by the phagocytes in our system remains to be determined.

Blockade of IL-6 conferred protective properties upon aAC. IL-10 production did not differ between resting and aACs, suggesting that IL-10 does not contribute to the regulation of inflammation in this setting. Indeed, it is unclear how the ongoing inflammation avoids the suppressive effects of IL-10, but it is possible that IL-10 may be required for the suppressive effects of TGF-β to prevail. Although overproduction of IL-6 has been associated with resistance to the actions of IL-10 (29) and promotion of Th17 when TGF-β is present (30), it is possible that production of both IL-10 and TGF-β could overcome the actions of IL-6. Notwithstanding the role of IL-10, our data indicate that the balance between TGF-β and IL-6 orchestrates the immune response driven by DCs in response to ACs.

aACs induced only a very short burst of IL-6, which was sufficient to abolish the protective TGF-β response. It is tempting to speculate that if aACs were continuously generated rather than administered for only a short period, as was the case in this study, this IL-6 response could be sustained, leading to worsening inflammation. This scenario may occur during an infection resulting in a sustained production of TGF-β and IL-6, thereby increasing Th17 cells. Infected neutrophils that undergo apoptosis are known to promote inflammation via the induction of Th17 cells because of their ability to induce TNF-α, IL-6, and TGF-β production by phagocytes (11).

A number of reports have shown that DCs can induce B cells with suppressive properties, although these have been associated with IL-10–producing B cells (31, 32). TGF-β production has only rarely been associated with suppression mediated by B cells (33), but therapeutic IL-6 blockade in patients with rheumatoid arthritis leads to an increase in regulatory B cells producing TGF-β (34).

Our findings could have important implications regarding the links between infection and autoimmunity. Infected ACs may either initiate inflammation and autoimmunity or exacerbate disease when patients with autoimmunity suffer concomitant infections. It is tempting to speculate that targeting IL-6, which is already available in the clinic, may reduce the severity of inflammatory disease during infective episodes and/or reduce the tendency of infections to trigger an exacerbation of inflammation. In patients with systemic lupus erythematosus, the production of autoantibodies targeting dsDNA and other nuclear Ags are thought to arise as ACs are inadequately cleared by phagocytic cells (5). However, our data suggest that ACs under certain states of activation may contribute to the worsening disease. Therefore, enhancing the efficiency of their removal without controlling inflammation could be detrimental. Similarly, concurrent infections may also exacerbate disease. A multitiered approach may be optimal involving treatment of infection, control of inflammation, and finally adequate removal of ACs.
